# Referral Activity in Three Store-and-Forward Networks during the COVID-19 Coronavirus Pandemic

**DOI:** 10.1155/2021/6644648

**Published:** 2021-05-03

**Authors:** Richard Wootton, Hansel Otero, Meghan Moretti

**Affiliations:** ^1^Collegium Telemedicus, Somerset TA1 1EG, UK; ^2^Children's Hospital of Philadelphia, Philadelphia, PA 19104, USA; ^3^The Addis Clinic, Nashville, TN 37212, USA

## Abstract

We surveyed three well-established store-and-forward telemedicine networks to identify any changes during the first half of 2020, which might have been due to the effect of the COVID-19 coronavirus pandemic on their telemedicine operations. The three networks all used the Collegium Telemedicus system. Various quantitative performance indicators, which included the numbers of referrals and the case-mix, were compared with their values in previous years. Two of the three networks surveyed (A and B) provided telemedicine services for any type of medical or surgical case, while the third (network C) handled only pediatric radiology cases. All networks operated in Africa, but networks A and C also provided services in other resource-constrained regions. Two of the networks (networks B and C) used local staff to submit referrals, while network A relied mainly on its expatriate staff. During the first half of 2020, the numbers of referrals received on network B increased substantially, while in contrast, the numbers of referrals on network A declined. All three networks had relatively stable referral rates during 2018 and 2019. All three networks delivered a service that was rated highly by the referrers. One network operated at relatively high efficiency compared to the other two, although it is not known if this is sustainable. The networks which were more reliant on local referrers saw little reduction—or even an increase—in submitted cases, while the network that had the most dependence on international staff saw a big fall in submitted cases. This was probably due to the effect of international travel restrictions on the deployment of its staff. We conclude that organizations wanting to build or expand their telemedicine services should consider deliberately empowering local providers as their referrers.

## 1. Introduction

Collegium Telemedicus is a not-for-profit organization, which provides free technical support to groups conducting humanitarian or noncommercial telemedicine services in low resource settings. The organization offers the infrastructure necessary to set up and operate a store-and-forward telemedicine service. The Collegium system is provided under a software-as-a-service model and is designed to be easy to use and to be able to service requests from increasing numbers of users (see https://collegiumtelemedicus.org for further details).

The Collegium system was first made available in 2012, and over the subsequent six years, 46 networks were established. The majority of the networks were set up to provide a clinical service (72%), with six networks (13%) designed for education and training, and the remainder (15%) for test or administrative purposes [1]. The two most active networks handled almost 12,000 cases during that period.

Since the initial review conducted in 2018, the Collegium system has continued to be widely used by different organizations. While some networks ceased their activity when their telemedicine work came to an end, new networks have begun operations. At the time of writing (July 2020), over 23,000 cases have been managed by the networks.

In December 2019, a new severe acute respiratory syndrome coronavirus 2 was identified. It began to spread rapidly around the world and the World Health Organization (WHO) declared the outbreak a global pandemic in March 2020. Although the case fatality rate of SARS-CoV-2 is lower than that of SARS-CoV and MERS-CoV, COVID-19 has resulted in more fatalities than other coronaviruses [2]. Major infection-control measures were put into place around the world during 2020 in an effort to reduce the spread of the disease. Between March and April 2020, over nine-tenths (91%) of the world's population were living in countries with travel restrictions, and over half of the world population was under some form of stay at home/quarantine order [3].

Against this background, several of the well-established Collegium networks began to prepare for the effect of the pandemic on their telemedicine operations in early 2020. For example, one network operated by a large humanitarian organization anticipated that the numbers of telemedicine cases would increase rapidly, and that additional resources, such as extra case coordinators and specialist expertise, would be required. Other networks were more sanguine about the size of any expected change in their numbers of telemedicine cases.

Organizational changes in advance of an expected global event are likely to have resource implications: more resources expended on telemedicine are likely to mean fewer resources for conventional methods of health care. Yet there is little published information about telemedicine planning for expected global events. The closest report of which we are aware concerns the planning for a telemedicine minor-injuries network to support expected patient numbers during a solar eclipse in 1999 [4].

Because of the dearth of information about the likely effect of the coronavirus pandemic, we surveyed three well-established telemedicine networks to assess any changes during the first half of 2020 compared to previous years.

## 2. Methods

We examined three Collegium telemedicine networks (networks A, B, and C) which were managing clinical cases from low-resource settings during 2020 and had been active for at least three years. In accordance with the Conditions of Use of the Collegium system (https://www.collegiumtelemedicus.org/ct/conditions.php?lang=en), only those networks which provided permission are identified here. We analysed various characteristics of their operation under eight main headings:

1. Network characteristics
2. Network demand
3. Case mix
4. Case complexity
5. Case management
6. Network performance
7. Network resources
8. Network efficiency

In each area, various quantitative performance indicators were evaluated. For example, case mix was defined in terms of the types of queries (i.e., the expertise of the specialists who were consulted) involved in the management of the cases. Case complexity was approximated by the numbers of messages per case, the numbers of queries per case, the proportion of unanswered queries, and the dialogue time. Case management was defined in terms of various performance statistics, such as the allocation delay. See Table 1 for a full description.

One important index of network demand is the referral rate, i.e., the number of new cases submitted in unit time. The referral rate was calculated for each network, for the months January to June 2020. The baseline, which was used as a comparator, was the average of the monthly rates from January to June 2018 and January to June 2019 in the same network.

We analysed the performance of each network in terms of the number of cases received and the delay in answering them. In addition, we examined the user feedback which had been provided by the referrers in the course of their use of the system. Three weeks after each case was submitted, the referrer was automatically sent an invitation to complete a multiple choice questionnaire about the case. The questionnaire contained 12 questions, of which three in particular were relevant to network performance:

Q6 “Did you find the advice helpful?” (yes/no/do not know)

Q8 “Do you think the eventual outcome for the patient will be beneficial?” (yes/perhaps/no/do not know)

Q9 “Was there any educational benefit to you in the reply?” (yes/no)

Finally, we examined the network efficiency, in terms of the outputs produced and the input resources consumed. Making the assumption that each case submitted to a network was dealt with to the satisfaction of the referrer, for which there is some evidence [5], then the “output” of the network can be taken to be the number of cases that was managed by it during the study period. To handle a telemedicine case, the networks depended on case coordinators for overall case management, and panels of specialists who provided expertise to the referrers, i.e., these represent “input” resources. The network efficiency can be approximated as the output produced divided by the input resources.

## 3. Results

### 3.1. Network Characteristics

Two of the three networks surveyed (A and B) provided telemedicine services for any type of medical or surgical case, while the third (network C) handled only pediatric radiology cases. All networks operated in Africa, but networks A and C also provided services in other resource-constrained regions. Two of the networks (networks B and C) used local staff to submit referrals, while network A relied mainly on its expatriate staff, who were based at the hospitals concerned for short periods (Table 2). Two of the networks (networks A and B) employed staff to act as case coordinators, while network C relied on volunteers.

### 3.2. Network Demand

During the first half of 2020, there were 319 referrer accounts on network A, 257 on network B, and 15 on network C. The proportion of these referrers who actually submitted a case during the first half of 2020 was 43%, 23%, and 20%, respectively, see Table 3.

There were 640 potential referring sites on network A, 135 on network B, and 21 on network C. The proportion of sites from which cases had actually been submitted was 18%, 31%, and 14%, respectively.

During the first six months of 2020, a total of 1203 cases was received on Network A. The rates in previous years were somewhat higher, Table 4. In contrast, the referral rates on both networks B and C were higher in 2020 than in previous years.

### 3.3. Case Mix

**Figure 1:**
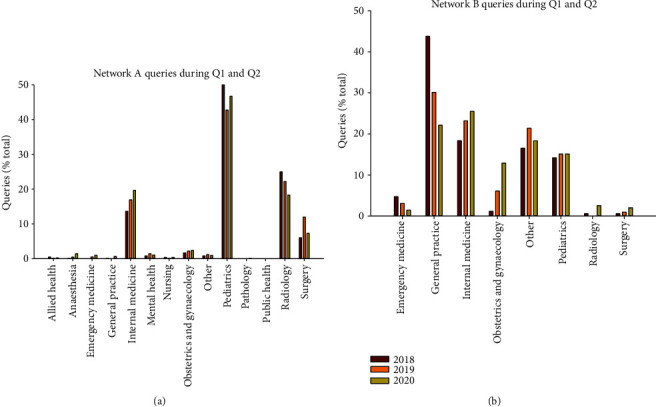
(a) Types of query for the cases on network A for the first six months of 2018, 2019, and 2020. (b) Types of queries for the cases on network B for the first six months of 2018, 2019, and 2020.

The two general networks, A and B, handled a wide range of query types. In 2020, the most common type of case managed on network A was pediatrics (47%, Figure 1(a)), while the most common category on network B concerned internal medicine (36%, Figure 1(b)).

There was some year-to-year variation in the query types. The mean ages of patients on network A were 21.1 years (*n* = 1176) and were 30.6 years (*n* = 844) on network B.

In contrast, network C, which handled only pediatric radiology cases, had a case mix that was exclusively radiology, both in 2020 and in previous years. The mean age of the patients was 5.7 y (*n* = 58), see Table 5.

On network A, there were approximately equal numbers of male and female patients, while on network B, there were more females (57%), and on network C, there were more males (62%).

### 3.4. Case Complexity

The mean number of messages per case on network A was 12.4. In contrast, the mean number of messages per case was lower on networks B and C (5.2 and 5.4, respectively).

The same pattern was observed for the mean number of queries per case, the mean number of unanswered queries, and the dialogue time—all were similar between network B and C, but much higher on network A (Table 6).

### 3.5. Case Management

Cases were allocated manually on networks A and B (100% of cases and 99.9% of cases), while on network C, only 40% of cases were allocated manually, i.e., 60% of cases were allocated automatically without requiring intervention from the case coordinator. The mean allocation delay was lower on networks A and B (0.23 and 0.70 h, respectively) than on network C (5.6 h). The mean numbers of messages sent by coordinators were highest on network A at 5.6 per case. It was lower on networks B and C (2.2 and 1.2, respectively) (Table 7).

### 3.6. Network Performance

The mean answer delay on network A was 17 h (SD 29, *n* = 1186). The mean answer delays on networks B and C were longer, at 53 h (SD 96, *n* = 769) and 24 h (SD 33, *n* = 59), respectively.

The proportion of the user-feedback questionnaires completed by the referrers was 17% on network A, 13% on network B, and 31% on network C. The majority of respondents (95% or higher) reported that they had found telemedicine advice to be useful. The respondents were more cautious about predicting a beneficial outcome for their patient following the telemedical interaction: 65-96% of respondents answered yes or perhaps. A higher proportion, 85-95%, felt that the telemedicine replies had been of educational benefit to them personally, see Table 8.

### 3.7. Network Resources

In network A, 416 specialists were available to respond to queries during the first half of 2020, of whom 80% were actually sent a query. There were fewer specialists available in networks B and C (120 and 35, respectively), but a roughly similar proportion were actually sent a query about a case (69% and 60%), see Table 9.

In network A, there were 25 case coordinators available during the first half of 2020, of whom about half (14) actually allocated queries. In networks B and C, there were fewer case coordinators available (6 and 3, respectively), but a roughly similar proportion of them actually allocated queries (50% and 33%).

### 3.8. Network Efficiency

For various reasons, not all case coordinators managed cases, and not all specialists were asked to respond to cases, during the study period. In networks A and C, the case coordinators managed similar numbers of cases, 86 and 65 per coordinator. In network B, the number of cases managed per coordinator was about four times higher, at 285, see Table 10.

Also, in networks A and C, the number of cases sent to each specialist was similar, at 3.6 and 3.1. In network B, the number of cases sent to each specialist was about three times higher, at 10.3.

Network B therefore appeared to be working some 3-4 times more efficiently than networks A and C.

### 3.9. Changes in Referral Rates during 2020

All three networks had relatively stable referral rates during 2018 and 2019. During the first half of 2020, the numbers of referrals received on Network B increased substantially, while in contrast, the numbers of referrals on network A declined (Table 11).

**Figure 2:**
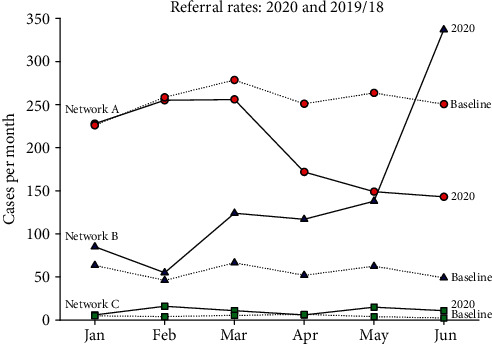
Referral rates in the three networks surveyed. Numbers of cases per month are shown for the year 2020 (solid lines), while the baseline values indicate the average numbers of cases during the corresponding months in 2019 and 2018 (dotted lines).

The steep rise in referral rates on network B occurred in May 2020, against a relatively constant baseline rate. The fall in referral rates on network A occurred during the second quarter of 2020, against a relatively constant baseline rate (Figure 2).

## 4. Discussion

There has been little published information on the effect of the COVID-19 coronavirus pandemic on telemedicine referral rates in low-resource settings. In May 2020, Helou et al. surveyed physicians in Lebanon who reported a modest increase in the use of WhatsApp, telephone calls, and email for telemedical purposes [6]. We have studied referral rates in three well-established telemedicine networks—formal networks—during the first half of 2020 and also in the two preceding years.

### 4.1. Similarities between the Networks

There were some similarities between the networks, in the sense that they delivered telemedical management advice to referrers based in Africa and elsewhere, using specialists many of whom were volunteers. There were also similarities in the way that the referrers rated the quality of the teleconsultations. On all three networks, 95% or more of responders rated the advice they received as helpful. On all three networks, 85% or more of responders rated the advice they received as being of educational benefit to themselves. A slightly lower proportion, 65% or more of responders, felt that the eventual outcome for the patient was likely to be beneficial. These perceptions are similar to those in larger studies [7]. That is, the principal users of the service found that the telemedicine advice they received was helpful, changed diagnosis and management, and/or reassured the patient.

### 4.2. Differences between the Networks

Despite the similarities, there were also differences between the networks in terms of their basic characteristics, such as the resources available to them, i.e., numbers of specialists and numbers of case coordinators. Network C was a relatively small network, specialising in pediatric radiology, while networks A and B were much larger and handled cases of a general nature. Network A was operated by a large international organization, and the resources available such as the numbers of case coordinators and specialists were some 3-4 times more than those available to network B.

Perhaps because network C was relatively less well-resourced than the others and relied on volunteer case coordinators, allocation delay was longer than on networks A and B.

Resources may also explain the differences between the networks in their average length of time between the submission of a case and the first response from a specialist. The answer delay was lowest in network A during 2020 and greater in networks B and C. Nonetheless, the answer delays were similar to those reported in other large networks such as that of Brazil [8] and French Guiana [9].

The number of queries per case remained largely constant on all three networks during the three years studied. However, they were rather different between the networks—more than twice as many queries per case for network A than for network B (Table 6). This could mean that the cases on network A were more complex and therefore required more specialist opinions, or it might simply reflect a different workflow that was more bureaucratic and required input from a larger number of team members. There was some evidence for the latter, since the proportion of unanswered queries was higher on network A, i.e., this could be interpreted as administrative colleagues being copied into the dialogue about a case. On the other hand, there were other indicators of complexity in the case mix on network A, and the average age of the patients was lower (16.9 years on network A vs. 30.3 years on the network B). So increased case complexity may explain, at least partly, why the number of queries per case was much higher in network A than in the others.

There were also differences between the networks in terms of their efficiency. One measure of network efficiency is the number of cases per coordinator, which on network B was about three times higher than on network A. Similarly, the number of cases per specialist was some three times higher on network B (Table 10). This could indicate that network B was operating more efficiently. Nonetheless, network A achieved rapid case allocation, and its average delay in providing the first response to the referrer was lower than the other networks, so in these respects, the quality of the service provided was relatively higher. Furthermore, it is not known whether high network efficiencies, which are reflected in higher case loads per specialist, are sustainable in the longer term.

### 4.3. Changes in Referral Rates in 2020

Telemedicine referral rates in the first half of 2020 were compared with those in previous, prepandemic years. Activity on network C, a small network specialising in pediatric radiology, remained in 2020 much as it had done before. However, substantial differences were observed in the referral rates on the two general networks, A and B.

In network B, referral rates increased substantially during 2020. This network had expanded its referral network during 2020 by increasing the number of staff in Kenya. These contracted clinical officers served as ambassadors who educated, trained, and supported fellow clinical officers in the use of the telemedicine services offered by the network in rural Kenya. In January 2020, there was one such individual, but by June 2020, there were five. The coronavirus pandemic did not affect this work because the staff members remained local and were not subject to any travel restrictions imposed at the international level.

**Figure 3:**
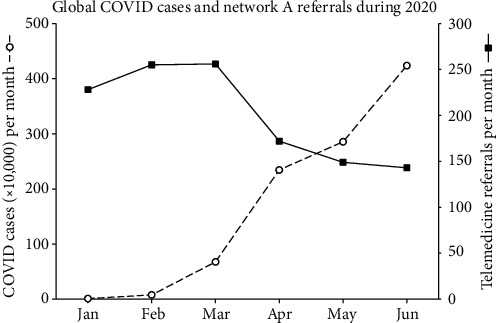
Global COVID cases during the first half of 2020 and the referral rate on network A.

In the other general network, however, referral rates fell during the first half of 2020. The reduction occurred specifically during the second quarter of 2020 and coincided with the increase in global COVID-19 cases. For convenience, the incidence of COVID-19 cases during 2020 (based on data from the WHO [10]) is plotted alongside the referral rate for network A in Figure 3.

The decline in referrals corresponds closely with the increase in global COVID-19 cases. Since telemedicine services in network A were heavily reliant on expatriate staff, it is unsurprising that international travel restrictions adversely affected the number of international staff who could be deployed. This in turn can be presumed to have driven the decline in their telemedicine referral rates.

Although the pandemic represents an obvious reason for the changes observed, additional factors should be considered. For example, it is possible that the nature of the telemedicine cases on network A changed during 2020, perhaps being restricted to more complex cases than before. However, although the number of cases decreased, the types of specialists required to manage them did not. Neither was there any substantial change in the case management, in terms of allocation delay or overall case-dialogue time. Therefore, the differences in the reliance on local referrers versus deployed expatriates probably explain much of the observed decline in the referral rates. This suggests that the development of sustainable telemedicine in low-resource settings is likely to rest on proper “stakeholder engagement,” a factor, which is frequently identified as important in studies of the readiness for e-health operations in developing countries [11].

## 5. Conclusion

The three networks studied operated in rather different ways, with differing resources available to them. All three networks delivered a service that was rated highly by the referrers. One network operated at relatively high efficiency compared to the other two, although it is not known if this is sustainable.

Our findings show that there were changes in the telemedicine service demand during 2020, which were very different in the three networks studied. Networks which were more reliant on local referrers saw little reduction—or even an increase—in submitted cases, while the network that had more dependence on international staff saw a big fall in submitted cases. This was probably due to the effect of international travel restrictions on the deployment of staff.

We conclude that organizations wanting to build or expand their telemedicine services should consider deliberately empowering local providers as their referrers. Not only will this reduce the fragility of the service when international travel restrictions are imposed but it might be politically advantageous to the organization in the sense of allowing it to leave behind a virtual presence when their field staff is withdrawn at the end of a finite project. That is, the legacy of the work on the ground could be a “virtual hospital” which continues to be supported by the organization concerned.

## Figures and Tables

**Table 1:** Principal indicators used in the present paper.

Area	Indicator	Comment
Network characteristics	Type of organization	
	Main purpose	
	Types of case	
	Countries of operation	

Network demand	Number of potential referrers	
	Referrers who had submitted a case	
	Number of potential referring sites	
	Referring sites from which a case had been submitted	
	Referrals submitted	

Case mix	Three most common query types	The type of a query is defined by the specialty and subspecialty of the specialist to whom it is sent
	Mean patient age in years (SD)	
	Sex (% male, % female)	

Case complexity	Number of messages per case	The total number of messages for the case
	Number of queries per case	The total number of queries sent to specialists
	Proportion of unanswered queries (%)	The proportion of the queries sent which were not answered
	Dialogue time (h)	The dialogue time is the interval (h) from case receipt until the last message from the referrer or specialist (excluding any progress report)

Case management	Proportion of cases allocated manually	The proportion of cases that were not allocated automatically (i.e., by the computer) to specialists
	Allocation delay (h)	The allocation delay is a measure of the performance of the coordinator(s) during the period in question. Every case will result in at least one query. The interval between the arrival of the case and the first time it is allocated for reply represents the allocation delay. This is true even if say a case results in two queries, and the first goes unanswered. The allocation delay, which is measured in hours, is defined as the delay before the first query was sent out, irrespective of whether that query was actually answered. If automatic allocation is in use, then cases on which it is used will have zero allocation delay.
	Number of coordinator messages per case	The number of messages sent by the coordinator(s)

Network performance	Answer delay (h)	The answer delay is a general measure of network performance, as perceived by the referrer. The answer delay, which is measured in hours, is defined as the delay after a case has been submitted before the first reply is received from a specialist. If queries are sent to several specialists (e.g., if the case is allocated to an expert group), then, the answer delay is measured from case submission to the earliest reply received.
	Number of completed questionnaires	
	Proportion of questionnaires completed (%)	
	Q6 “did you find the advice helpful?” (% yes)	
	Q8 “do you think the eventual outcome for the patient will be beneficial?” (% yes)	
	Q9 “was there any educational benefit to you in the reply?” (% yes)	

Network resources	Specialists available	
	Specialists who were sent a query during 2020 (% of those available)	
	Specialists who answered a query during 2020 (% of those sent a query)	
	Case coordinators available	
	Case coordinators who allocated a query during 2020 (% of those available)	

Network efficiency	Cases per potentially-available case coordinator	
	Cases per actual case coordinator	
	Cases per potential specialist	
	Cases per actual specialist	

**Table 2:** Characteristics of the three Collegium networks studied.

	Network A	Network B	Network C
Network identifier	22	42	25
Operator	International humanitarian organization	The Addis clinic	World Federation of Pediatric Imaging
Main purpose	Clinical case support for hospital staff mainly provided by the organization itself	Clinical case support for local hospital staff	Clinical case support for local hospital staff
Types of case	General	General	Radiology
Countries	Many, mainly in Africa and Asia	Kenya, Cameroon, and Ethiopia	Mozambique, South Africa, and Laos

**Table 3:** Network demand during the first six months of 2020.

	Network A	Network B	Network C
Number of potential referrers	319	257	15
Referrers who had submitted a case	136	58	3
Number of potential referring sites	640	135	21
Referring sites from which a case had been submitted	117	42	3
Referrals submitted	1203	856	65

**Table 4:** Referral rates during the first six months of 2018-2020.

Year	Network A	Network B	Network C
2018	1540	131	24
2019	1516	548	30
2020	1203	856	65

**Table 5:** Case-mix in the first six months of 2020.

	Network A	Network B	Network C
Three most common query types in 2020	Pediatrics (47%); internal medicine (20%); radiology (18%)	Internal medicine (36%); general practice (22%); pediatrics (15%)	Radiology (100%)
Mean patient age in years (SD)	21.1 (20.5)	30.6 (21.5)	5.7 (8.1)
Sex (%M, %F)	51, 47	43, 57	62, 39

**Table 6:** Message activity in the first six months of 2020.

	Network A	Network B	Network C
Number of messages per case (SD)	12.4 (8.2)	5.2 (1.8)	5.4 (2.4)
Number of queries per case (SD)	2.8 (1.6)	1.1 (0.5)	1.2 (0.5)
Number of unanswered queries per case (SD)	0.67 (0.96)	0.18 (0.45)	0.28 (0.51)
Dialogue time in hours (SD)	280 (451)	85 (144)	71 (274)

**Table 7:** Case management in the first six months of 2020.

	Network A	Network B	Network C
Percentage of cases allocated manually	100	99.9	40
Allocation delay in hours (SD)	0.23 (3.46)	0.70 (4.53)	5.56 (3.10)
Number of coordinator messages per case (SD)	5.6 (3.2)	2.2 (0.6)	1.2 (1.3)

**Table 8:** User feedback: responses to three of the 12 questions.

	Network A	Network B	Network C
Number of questionnaires completed	208	109	20
Proportion of questionnaires completed (%)	17	13	31
Q6 “did you find the advice helpful?” (% yes)	95	96	100
Q8 “do you think the eventual outcome for the patient will be beneficial?” (% yes)	52	93	20
Q8 “do you think the eventual outcome for the patient will be beneficial?” (% yes or perhaps)	76	96	65
Q9 “was there any educational benefit to you in the reply?” (% yes)	89	95	85

**Table 9:** Resources (numbers of specialists and case coordinators) available during the first six months of 2020.

	Network A	Network B	Network C
Specialists available	416	120	35
Specialists who were sent a query during 2020 (% of those available)	331 (80)	83 (69)	21 (60)
Specialists who answered a query during 2020 (% of those sent a query)	268 (81)	75 (90)	13 (62)
Case coordinators available	25	6	3
Case coordinators who allocated a query during 2020 (% of those available)	14 (56)	3 (50)	1 (33)

**Table 10:** Network efficiency during the first half of 2020.

	Network A	Network B	Network C
Cases during 2020	1203	856	65
Cases per potentially-available case coordinator	48	143	22
Cases per actual case coordinator	86	285	65
Cases per potential specialist	2.9	7.1	1.9
Cases per actual specialist	3.6	10.3	3.1

**Table 11:** Referral rates (cases per quarter) in the three networks during the first half of 2020.

	Q1 2020	Q2 2020	Difference
Network B	264	592	328
Network C	33	32	-1
Network A	739	464	-275

## Data Availability

The data used to support the findings of this study are available from the corresponding author upon request, subject only to consideration of any legal and ethical concerns.
